# Volatile Organic Compound-Based Predictive Modeling of Smoke Taint in Wine

**DOI:** 10.1021/acs.jafc.3c07019

**Published:** 2024-03-27

**Authors:** Cheng-En Tan, Bishnu Prasad Neupane, Yan Wen, Lik Xian Lim, Cristina Medina Plaza, Anita Oberholster, Ilias Tagkopoulos

**Affiliations:** † Department of Computer Science, University of California, Davis, Davis, California 95616, United States; ‡ Genome Center, University of California, Davis, Davis, California 95616, United States; § USDA/NSF AI Institute for Next Generation Food Systems (AIFS), University of California, Davis, Davis, California 95616, United States; ∥ Department of Viticulture and Enology, University of California, Davis, Davis, California 95616, United States

**Keywords:** smoke taint, wine industry, volatile organic compounds, flavor, computational modeling

## Abstract

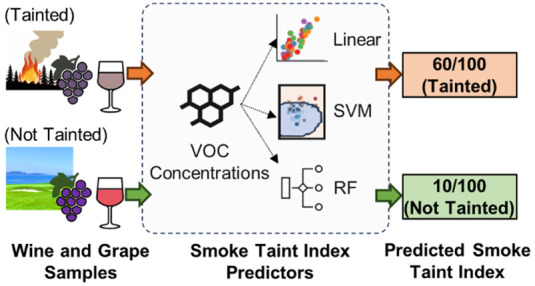

Smoke taint in wine has become a critical issue in the wine industry due to its significant negative impact on wine quality. Data-driven approaches including univariate analysis and predictive modeling are applied to a data set containing concentrations of 20 VOCs in 48 grape samples and 56 corresponding wine samples with a taster-evaluated smoke taint index. The resulting models for predicting the smoke taint index of wines are highly predictive when using as inputs VOC concentrations after log conversion in both grapes and wines (Pearson Correlation Coefficient PCC = 0.82; *R*^2^ = 0.68) and less so when only grape VOCs are used (Pearson Correlation Coefficient PCC = 0.76; *R*^2^ = 0.56), and the classification models also show the capacity for detecting smoke-tainted wines using both wine and grape VOC concentrations (Recall = 0.76; Precision = 0.92; F1 = 0.82) or using only grape VOC concentrations (Recall = 0.74; Precision = 0.92; F1 = 0.80). The performance of the predictive model shows the possibility of predicting the smoke taint index of the wine and grape samples before fermentation. The corresponding code of data analysis and predictive modeling of smoke taint in wine is available in the Github repository (https://github.com/IBPA/smoke_taint_prediction).

## Introduction

Bushfire and forest burn events may negatively impact the quality of wines which are described as “smoke tainted” with several unfavorable characteristics such as “smoke”, “burnt”, “ash”, and “ashtray”.^1,2^ The quality loss of grapes and wines due to smoke taint from bushfires can be substantial with losses amounting to hundreds of million dollars or more each year in Australia^3,4^ and the United States.^5^ Due to climate-induced weather changes such as temperature increase, drought, wind, and natural ignition sources,^6,7^ the incidence of significant forest fires reported in Europe,^8,9^ North America,^9^ Australia,^4^ and other regions across the globe^10^ is increasing, and it escalates the level of negative impact in the wine industry around the world.

During wildfires, several materials including smoke, substantial quantities of gases, and volatile organic compounds (VOCs) are released.^4^ These released materials are part of the products of the wood combustion process including heating, dehydration, hydrolyzation, oxidization, and pyrolyzation.^4,11,12^ Among these materials, VOCs are reported as the potential substance which may cause contamination of vines,^4^ and several studies show that the concentrations of VOCs are elevated in smoke-tainted wine^2,13,14^ and also show that the VOCs are correlated with undesirable smoky and ashy sensory characters.^1,14^ Due to the significant relationship between VOCs and smoke taint levels, different variants of studies related to VOCs in grapes and wine are published: These studies include mitigating the smoke taint effect by reducing VOC absorption and production before,^2,15−17^ during,^18^ and after fermentation,^19,20^ and observing the changes of VOC concentrations during fermentation.^6,21,22^

Finding VOCs that impact the smoke taint index can be useful, as they may help the fast and reproducible identification of tainted samples and the development of mitigation approaches. Data-driven approaches, especially machine learning, can accelerate the discovery of the VOCs related to smoke taint and the predictive modeling of the smoke taint index. In recent years, machine learning algorithms as a part of the Artificial Intelligence (AI) have increasingly been applied in food science and agriculture for a sustainable food system,^23^ including predicting micronutrients,^24−26^ creating food ontologies and knowledge bases,^27^ precision agriculture,^28^ and crop and animal management.^29^ Although VOCs in smoke-affected grapes and wine have been reported,^30^ the levels contributing to the smoke taint effect of VOCs have been evaluated,^14^ and a few studies that model the smoke flavor based on chemical composition have been published recently,^31^ the number of studies focusing on data-driven approaches, especially predictive modeling of smoke taint based on VOC concentrations, are still limited.

**Figure 1:**
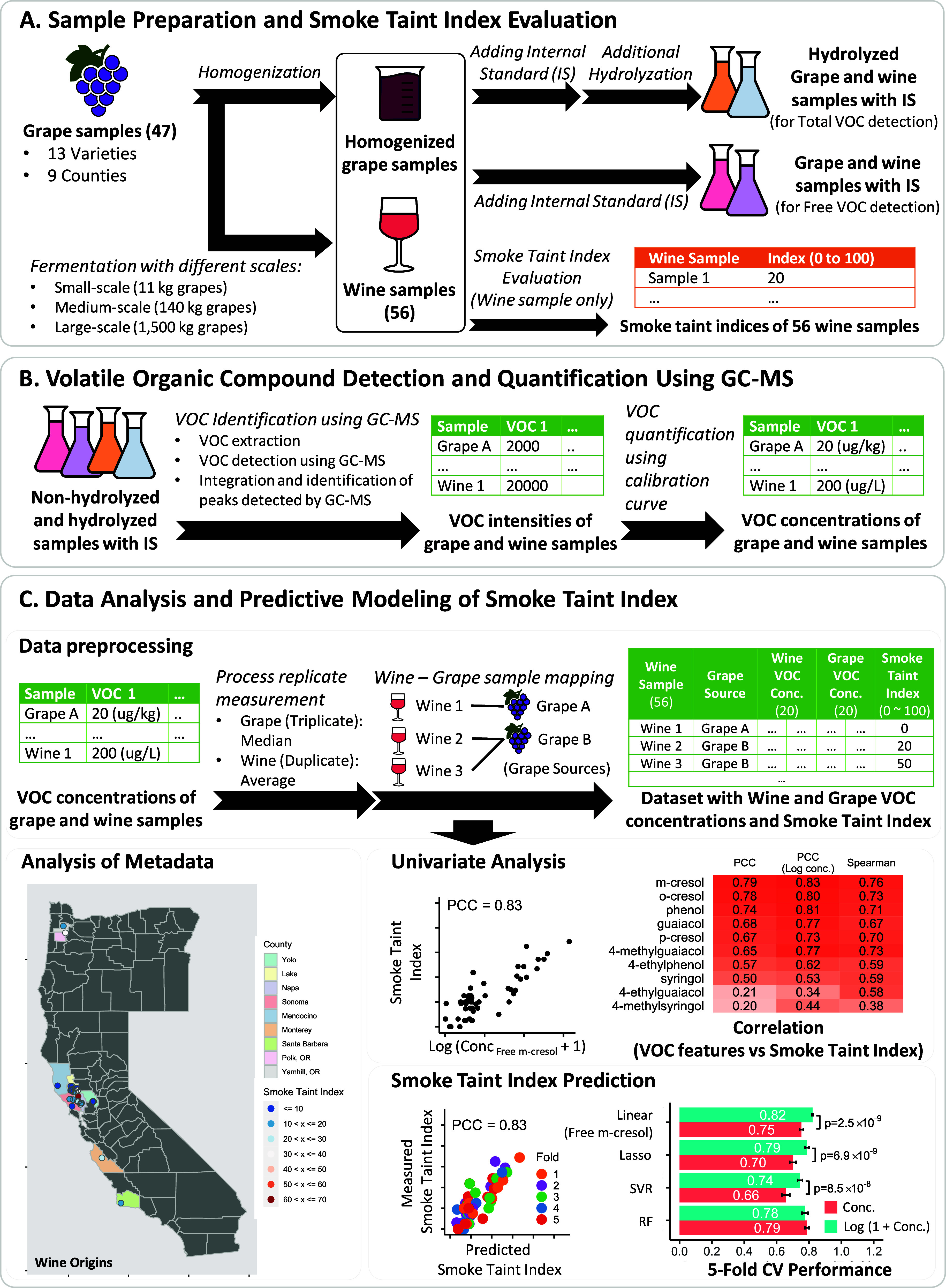
Flowchart of the sample preparation, volatile organic compound (VOC) quantification, data analysis, and predictive modeling of the smoke taint index. **A.** Sample preparation and smoke taint index evaluation by selected tasters. **B.** VOCs quantification. **C.** Data analysis and smoke taint index prediction.

In this study, we collected samples of 56 wines made from 47 grapes with 13 different varieties from 9 different counties in California and Oregon, which have been evaluated for smoke taint (Figure 1). We then applied machine learning techniques to create smoke taint predictors and identify the minimal set of compounds that can predict the presence of smoke taint, which resulted in the most informative combinations of compounds for each case.

## Data sets and Methods

### Sample Collection

**Figure 2:**
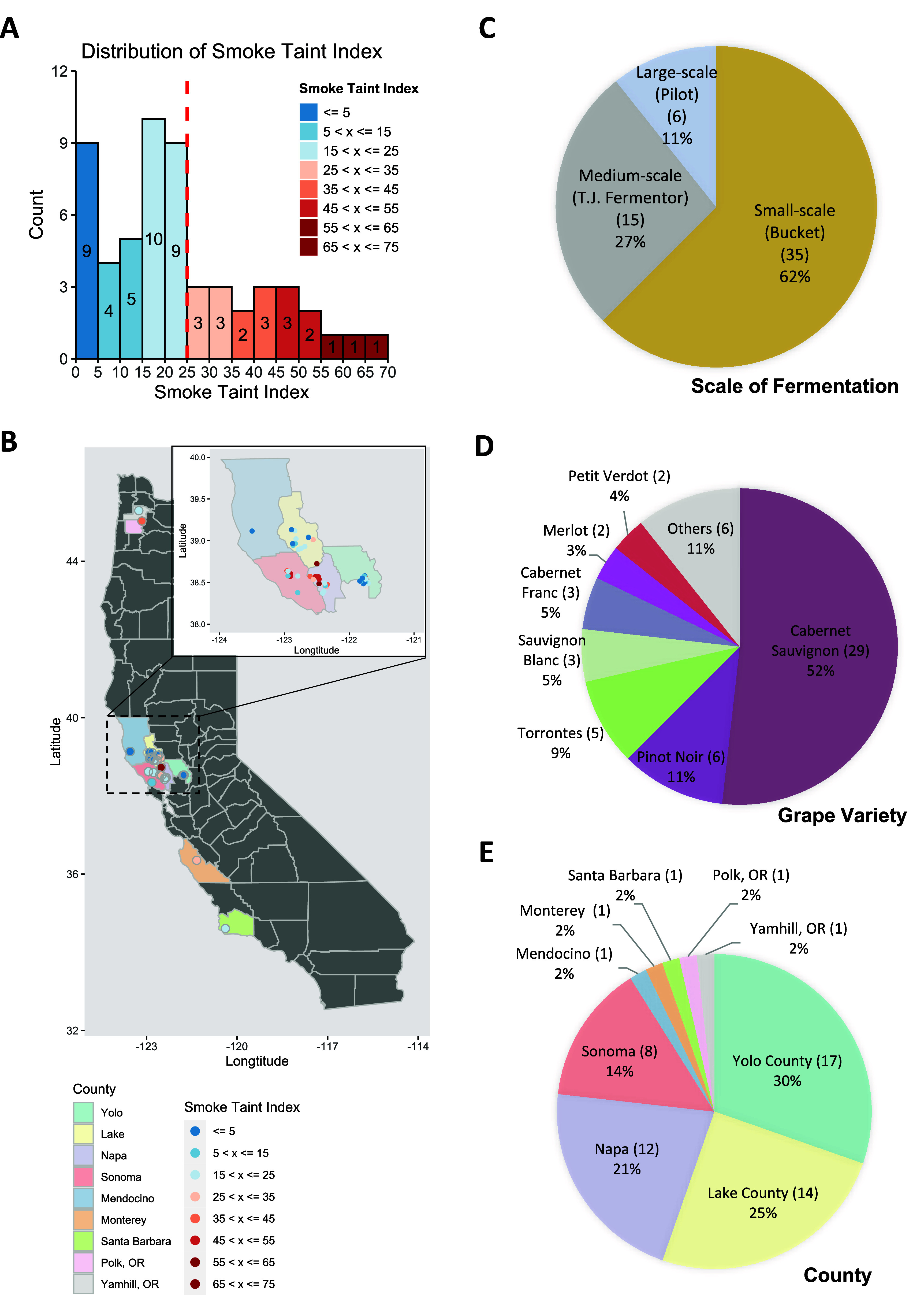
Description of the data set. **A.** Smoke taint index distribution of 56 wine samples. Nineteen samples with smoke taint index greater than 25 are considered as smoke-tainted. **B.** Origin of 56 wine samples colored by different smoke taint index levels. **C.** Distribution of fermentation scales. **D.** Distribution of 13 grape varieties of 56 wine samples. The other six varieties include Barbera, Grenache, Malbec, Petit Sirah, Syrah, and Zinfandel and each of them has one wine sample. **E.** Distribution of 9 counties in California and Oregon. The counties without additional specifications are in California.

The final data set contains the smoke taint indices of 56 wine samples made from 47 grape samples produced in 2020 (Figure 2A, Table S1) in California and Oregon (Figure 2B). Nineteen wine samples with a smoke taint index greater than 25 are considered as smoke-tainted. The wine samples were fermented in three different scales (Figure 2C) from 13 different varieties of grapes (Figure 2D) in nine counties (Figure 2E). The majority of wine samples are non-smoke-tainted with the smoke taint index no greater than 25 (37 samples, 66%), fermented with a lower scale (Bucket scale, fermentation with 11 kg grapes) (35 samples, 62%), and fermented from the grape with variety Cabernet Sauvignon (29 samples, 52%). In addition, more than 90% of wine samples (51 samples, 91%) are from four counties (Yolo, Lake, Napa, and Sonoma counties) in Northern California.

### Grape and Wine Sample Preparation and Volatile Organic Compound Extraction

For grape samples, an IKA digital ultraturrax (T18) disperser is used for homogenization, and then the internal standard solution which contains a mixture of eight reference compounds (d3-guaiacol, d3-4-methylguaiacol, d7-*o*-cresol, d7-*p*-cresol, d7-*m*-cresol, d5-4-ethylguaiacol, and d4-4-ethylphenol were obtained from CDN Isotopes (Pointe-Claire, QC, Canada) and d6-syringol was purchased from EPTES (Vevey, Switzerland)) with a concentration of 5 mg/L is added to homogenized grape samples and the wine samples (Table 1). For the case of extracting total VOCs, a harsh-acid hydrolysis method as described by Noestheden et al. with minor modifications was applied:^32^ Ten milliliters of homogenized grape and wine samples for the extraction of total VOCs spiked with internal standards (20 μg/L) is hydrolyzed by adjusting the pH of the samples to 1.0 using concentrated HCl and heated to 100 °C for 1 h.^33^ Recovery of all compounds was tested in three different matrixes (Cabernet Sauvignon, Pinot noir, and Merlot grapes) at two different concentrations (5 and 100 μg/kg) in triplicate. Recovery percentages of all compounds were between 83 and 126%, except for 4-methylsyringol (creosol) at 66% for free VOCs. For acid-labile VOCs the recovery percentages determined as described above were between 70 and 127%, except for 4-methysyringol (creosol) at 67% (manuscript in preparation). These results are very comparable with those of Noestheden et al.^32^ Finally, the free VOCs and the total VOCs were extracted as described in Oberholster et al.^33^ by adding the extraction solvent (the mixture of pentane and ethyl acetate with a ratio of 1:1) to the nonhydrolyzed and hydrolyzed samples, respectively (Figure 1A). After 10 min of extraction, centrifugation is then applied to VOC extraction mixtures, and the upper layer (organic layer) of the mixture is transferred for GC–MS/MS analysis.

**Table 1:** Targeted Volatile Organic Compounds (VOCs) and the Corresponding Internal Standards for Quantification

Targeted VOC	Precursor Ion (*m*/*z*)	Product Ion (*m*/*z*)	Retention Time (min)	Collision Energy (V)	Internal Standard (I.S.) referred	Precursor Ion (I.S.) (*m*/*z*)	Product Ion (I.S.) (*m*/*z*)	Retention Time (I.S.) (min)	Collision Energy (I.S.) (V)
guaiacol	123.9	109	9.058	10	d-guaiacol	127.1	109	9.039	10
guaiacol	123.9	81	9.058	20	d-guaiacol	127.1	81	9.039	20
4-methylguaiacol	138.1	123	9.811	10	d-4-methylguaiacol	141.1	126	9.799	10
4-methylguaiacol	138.1	95	9.811	20	d-4-methylguaiacol	141.1	98	9.799	20
*o*-cresol	108	107	10.146	15	d-*o*-cresol	115	113	10.146	20
*o*-cresol	108	77	10.146	15	d-*o*-cresol	115	81	10.146	30
phenol	94	66	10.201	10	d-4-ethylphenol	126.1	111	11.54	10
phenol	94	65	10.201	20	d-4-ethylphenol	126.1	80	11.54	30
4-ethylguaiacol	151.8	137	10.388	10	d-4-ethylguaiacol	157	139	10.34	10
4-ethylguaiacol	151.8	94	10.388	30	d-4-ethylguaiacol	157	96	10.34	30
*p*-cresol	108	107	10.801	15	d-*p*-cresol	115	113	10.752	20
*p*-cresol	108	77	10.801	15	d-*p*-cresol	115	85	10.752	20
*m*-cresol	108	107	10.87	15	d-*m*-cresol	115	113	10.821	20
*m*-cresol	108	77	10.87	15	d-*m*-cresol	115	85	10.821	20
4-ethylphenol	121.9	107	11.29	10	d-4-ethylphenol	126.1	111	11.29	10
4-ethylphenol	121.9	77	11.29	30	d-4-ethylphenol	126.1	80	11.29	30
syringol	153.9	139	12.266	5	d-syringol	160	142	12.227	10
syringol	153.9	65	12.266	20	d-syringol	160	114	12.227	20
4-methylsyringol	168	153	12.936	5	d-syringol	160	142	12.227	10
4-methylsyringol	168	125	12.936	10	d-syringol	160	114	12.227	20

### Volatile Organic Compound Quantification in Grape and Wine Samples

To detect and quantify the VOCs, targeted GC–MS/MS analysis was applied to grape and wine samples (Figure 1B). The VOCs were identified based on the precursor ion and retention time, and the VOCs were quantified based on the constructed calibration curve for each VOC with the range 0.25–500 μg/kg for grape samples and 0.25–500 μg/L for wine samples. The Limit of Detection (LOD) and the Limit of Quantitation (LOQ) of all compounds quantified were above 0.0649 and 0.1779 μg/L, respectively. LOD and LOQ were calculated as LOD = 3 × SD_ymin_/*S* and LOQ = 5 × LOD where SD_ymin_ is the standard deviation for the smallest calculated concentration and *S* is the slope of the respective regression. Triplicate and duplicate measurements are applied to grape and wine samples, respectively.

### Gas Chromatography–Mass Spectrometry Analysis

An Agilent 7890A gas chromatograph was coupled to an Agilent 7000B triple quadrupole mass spectrometer with an MPS 2 autosampler (Gerstel, Inc., Linthicum, MD). All peaks were integrated using MassHunter Qualitative Analysis software (ver. B.03.01, Agilent Technologies).

The gas chromatograph was fitted with a DB-WAXetr fused silica capillary column with dimensions of 30 m length × 0.32 mm i.d. × 1.0 μm film thickness (Agilent).

The inlet was held at 220 °C, while the oven program began at 75 °C and was held for 1 min followed by a 15 °C/min increase to 180 °C, followed by a 10 °C/min increase to 230 °C held for 1 min with another increase at 50 °C/min increase to 250 °C, held for 3 min. The total run time was 17.4 min. The interface between the GC and the MS was held at 220 °C. Samples were run in pulsed splitless mode; the split vent was opened at 1 min with a flow of 50 mL/min. Helium carrier gas was used at 2.0 mL/min in the constant flow mode. The triple quadrupole mass spectrometer was fitted with an electron ionization source operated at 70 eV.

The reagent gas was helium introduced to the source at a rate of 1 mL/min. The source temperature was 230 °C. The solvent delay was 7.5 min. Multiple reaction monitoring (MRM) quantitative and qualitative transitions and collision energies were chosen for each compound based on signal-to-noise ratios. Dwell times were set so that there were 15 scans over each peak to ensure quantitative peak integration. The nitrogen collision gas and helium quench gas was fixed at 1.5 and 2.25 mL/min, respectively.

### Smoke Taint Index Evaluation of Wine Samples

The smoke taint indices of the wine samples were evaluated by trained panelists. The “ashy” standard rating included in the descriptive analysis (DA) panels described in Oberholster et al.^33^ was applied for evaluating the smoke taint indices of all wine samples analyzed (Table S1). Descriptive analysis and subsequent consumer studies using serial dilution of smoke impacted wines determined that wines are considered “smoke tainted” when the “ashy” rating is >20 out of a 100. Wines made from grapes not exposed to smoke also obtained low “ashy” ratings in conducted studies.

### Data Preprocessing

The concentrations of VOCs in wine samples and the corresponding origin grape samples are merged for analysis. First, the average and median values are used to represent the VOC concentration of each sample for duplicate measurements (wine samples) and triplicate measurements (grape samples), respectively. Then two tables of VOC concentrations quantified in wine and grape samples are merged by mapping the corresponding grape samples for each wine sample. Different wine samples may be mapped to the same origin of grape samples. The final table contains concentrations of 20 VOCs in 56 wine samples and their corresponding grape samples (48 in total).

### Univariate Analysis and Feature Selection

To find the VOCs that are predictive of the smoke taint index, univariate analysis is performed by evaluating the correlation between the smoke taint index and the concentration of the VOCs in wine samples. In addition, feature selection is performed for predictive modeling by evaluating four different feature importance benchmarks including the loadings of the first components in Principle Component Analysis^34^ and Partial Least-Squares,^35^ the feature importance reported by Random Forest,^36^ and the order of feature selection reported by Sequential Forward Selection.^37^ The VOCs with at least two top-five rankings in these four benchmarks are selected for predictive modeling.

### Predictive Modeling

Regression models and classification models are built for predicting the smoke taint index in wine and detecting smoke-tainted wines using the selected features. For regression models, four different models (linear model using the VOC which are the most highly correlated with the smoke taint index, Lasso,^38^ Support Vector Regression,^39^ and Random Forest^36^) are applied for smoke taint index prediction. For classification, four different models (single VOC which is the most highly correlated with the smoke taint index, Logistic,^40^ Support Vector Machine,^41^ and Random Forest^36^) are applied. To evaluate the performance of models, 5-fold cross-validation (CV) is applied, and the Pearson Correlation between the predicted and measured smoke taint index and the classification performance (recall, precision, and F1-score) are evaluated. Ten repeats are applied, and the average 5-fold CV performances of four models are compared. Due to the observation that the correlation increases after applying the log conversion to VOCs concentration, the performances of the regression models using log-converted VOC concentrations as input features are also evaluated. In addition to models that use concentrations of VOCs in wine and their corresponding origin grape as input, the models that use concentrations of VOCs only in origin grape samples are also trained, and their 5-fold CV performances are also evaluated to observe the possibility of predicting smoke taint index before fermentation. All smoke taint prediction models are implemented in R programming language (version 3.6.1).^42^ For the Lasso model, the packet glmnet (version 4.1-1)^43^ is used and the searching range of λ is {10^–3^, 10^–2.9^, ..., 10^3^}. For the support vector regression model, the packet e1071 (version 1.7-13)^44^ is used and the searching range of cost, gamma, and epsilon are {10^–1^, 10^–0.9^, ..., 10^1^}, {10^–1.8^, 10^–0.7^, ..., 10^0.2^}, and {10^–2.2^, 10^–2.1^, ..., 10^–1.8^}, respectively. For the Random Forest model, the packet randomForest (version 4.6-14)^45^ is used and the searching range of mtry, node size, and the number of tree parameters are {0.25*N*, 0.5*N*, 0.75*N*, 1.0*N*}, {1, 3, 5, 10}, and {50, 100, 200, 500, 1000, 2000}, respectively (*N* is the number of input features, and *N* will be 20 for the model using only grape VOC concentrations as input or 40 for the model using both grape and wine VOC concentrations as input).

## Results

### Hierarchical Clustering Reveals the Consistent Location-Based Smoke Taint Pattern

**Figure 3:**
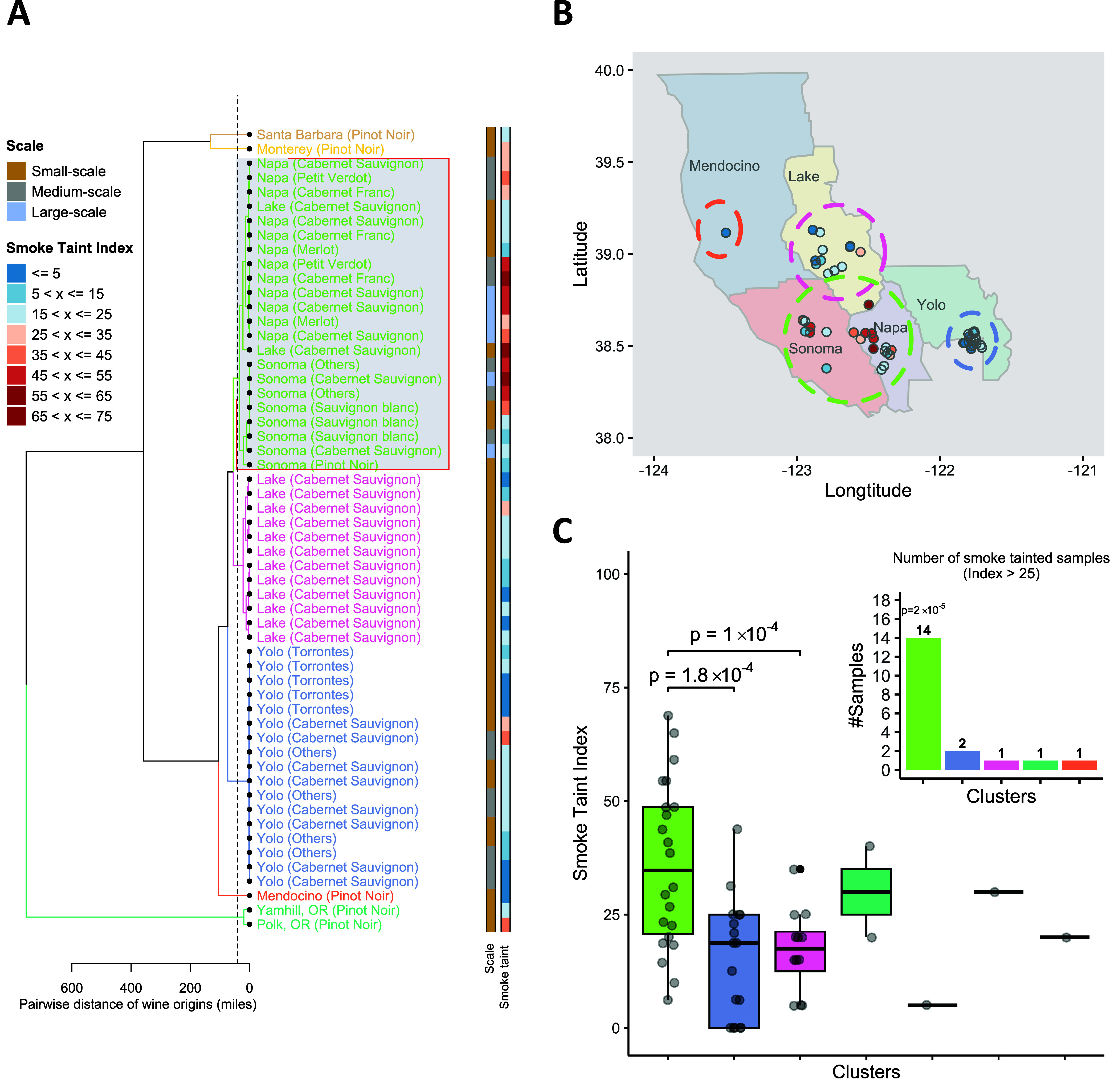
Relationship between the wine origin and the smoke taint index. **A.** Hierarchical clustering result of 56 wine samples. The clustering is based on the pairwise distance in miles. Seven clusters are extracted with the cutting threshold of 40 miles. **B.** Origin of wine samples from five counties in Northern California colored by different smoke taint levels and their corresponding clusters. **C.** Boxplot of the smoke taint index of the wine samples in seven clusters, and the number of smoke-tainted samples (with smoke taint index greater than 30) in four clusters that contains more than one wine sample.

We performed hierarchical clustering of the wine samples based on the pairwise geographical distances (in miles) among the origin of the wine samples. As expected, samples with high smoke taint scores are colocated geographically, with a clear cluster of those samples forming (green cluster, Figure 3A). The selected cluster contains wine samples from Sonoma and Napa and the border between Napa and Lake County (Figure 3B). The statistical results show that the smoke taint indices in the selected cluster are significantly higher than the smoke taint indices of wine samples from Yolo County (the cluster colored in blue) and Lake County (the cluster colored in pink). In addition, the hypergeometric test shows that the selected cluster has a significantly higher ratio of smoke-tainted wine samples (*p*-value = 2 × 10^–5^; Figure 3C).

### VOC Signatures Are Predictive of Smoke Taint in Wine Samples

**Figure 4:**
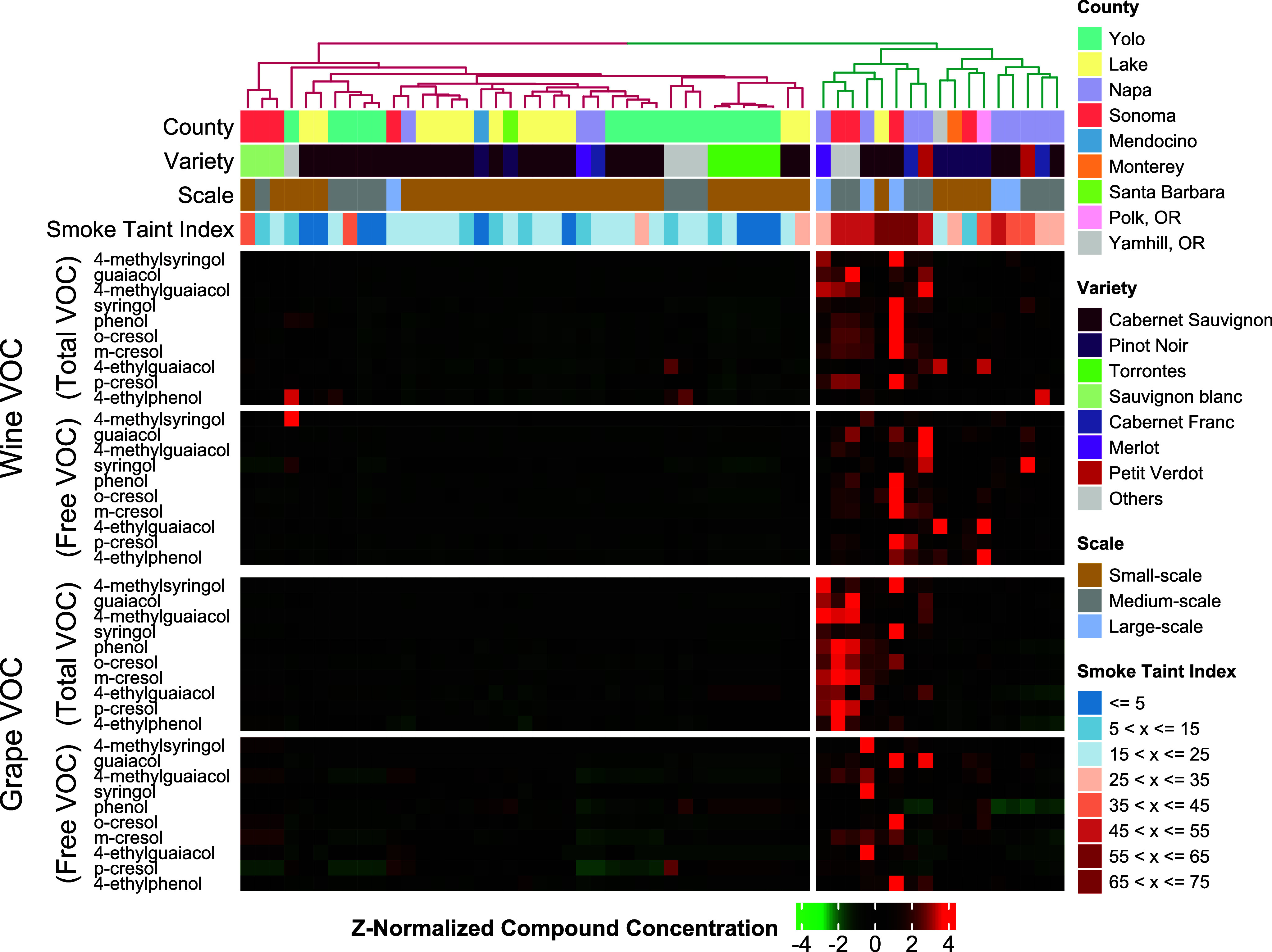
Z-Normalized VOC concentration of samples. Ten Total VOCs and 10 Free VOCs quantified in wine samples and their origin grape samples are shown.

Hierarchical clustering based on the VOC profiles including free and total VOC in wine and grapes argues that the smoke-tainted wine samples can be separated from non-smoke-tainted wine based on the VOC concentration distribution (Figure 4). As shown in Figure 4, the right cluster contains 17 wine samples, and 15 of them are smoke-tainted (with index >25), and the left cluster contains 39 wine samples, and only 4 of them are smoke-tainted. The hypergeometric test shows that the right cluster has a significantly higher ratio of smoke-tainted wine samples (*p* = 3.7 × 10^–10^). In addition, the concentrations of VOCs of wine samples in these two clusters are significantly different: The *t* test shows that 19 VOCs in wine samples and 12 VOCs in the corresponding origin grape samples have significantly higher concentrations in the right cluster (*p* < 0.05) compared with the VOC concentrations in the left cluster.

### Free *m*-Cresol in Wine and Total Syringol in Grapes Are the Most Predictive Indicators of Smoke Taint Index

**Figure 5:**
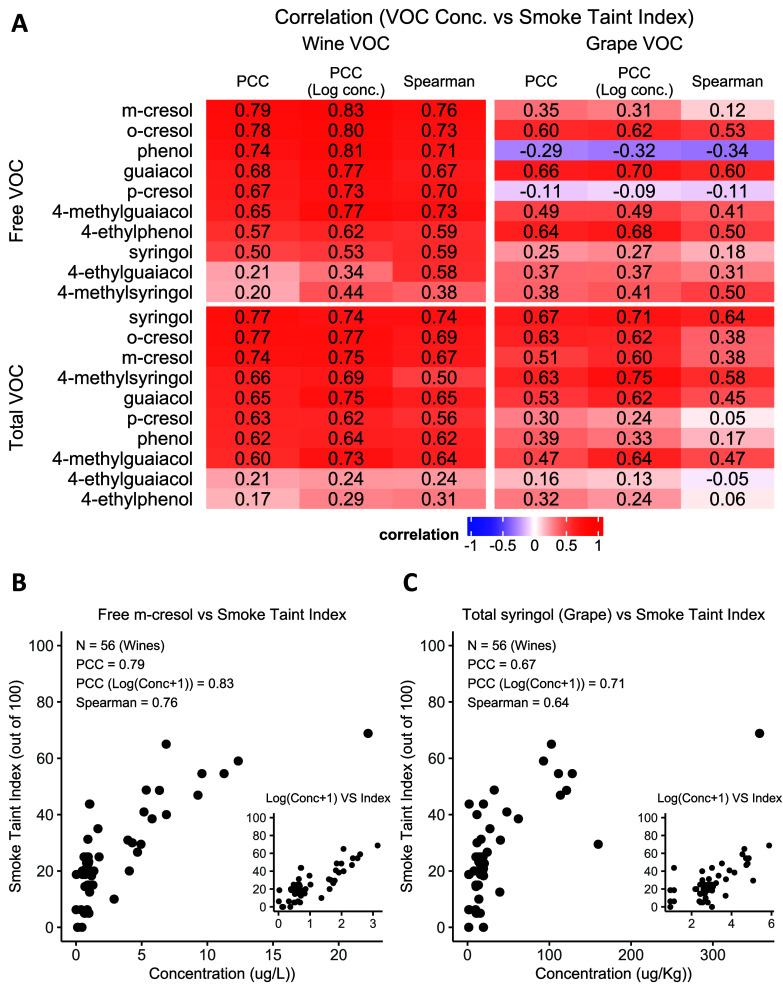
Univariate analysis results. **A.** Correlations between smoke taint index and VOCs in wine samples and their origin grape samples. **B.** Scatter plot of smoke taint index and free *m*-cresol concentration of wine samples. **C.** Scatter plot of smoke taint index of wine samples and total syringol concentration of their origin grape samples.

The predictiveness of the VOC features can be evaluated based on the correlations between the features and the smoke taint index (Figure 5A). For VOCs in wine samples and the corresponding grape samples, free *m*-cresol (in wine) and total syringol (in grapes) concentration are the most highly correlated with smoke taint indices with PCC 0.79 and 0.67, respectively (Figure 5B,C). In addition, the scatter plots show the nonlinearity between the VOC concentrations: As the VOC concentration increases, the slope of VOC concentrations and the smoke taint index decreases. For this reason, we performed log-normalization of the VOC concentration, which we found to be more predictive of the smoke taint index (PCC of 0.83 and 0.71 for *m*-cresol and total syringol, respectively; Figure 5A).

### Comparison of VOC Concentrations in Wine and Origin Grape Samples

Measurement of the VOC concentration in both wine samples and the corresponding origin grape sample allows us to observe the VOC composition changes after fermentation by comparing the composition ratio in wine and grape samples: The composition ratio of free syringol increased by 33.2% and 48.4% after fermentation for non-smoke-tainted and smoke-tainted wine samples, respectively (Figure S3). In addition, the pattern of VOC composition ratio changes can be compared in smoke-tainted wine samples and non-smoke-tainted wine samples: The average composition ratios of free phenol and free *p*-cresol decreased by 23.5% and 15.0% after fermentation in non-smoke-tainted wine, but the composition decreased by only 3.3% and 7.9% in smoke-tainted wine. In contrast, the average composition ratios of free guaiacol and free *o*-cresol decreased by more than 10% in smoke-tainted wine but only about 5% in non-smoke-tainted wine. Moreover, the composition ratio changes of free VOCs and total VOCs can also be compared: For phenol and guaiacol, the composition ratio of both free VOCs and total VOCs decreased during fermentation; for syringol, both free and total composition ratio increased; and for *o*-cresol and *p*-cresol, only the composition ratio of free VOCs decreased.

The correlations between VOC concentrations in wine samples and the corresponding origin grape samples may also reveal the VOC composition changes during fermentation (Figure S4). The patterns of the distribution of correlations are different in non-smoke-tainted wine and smoke-tainted-wine: For smoke-tainted wine samples, the concentrations of free 4-ethylphenol, total syringol, and total 4-methylsyringol are highly correlated to the VOC predictive smoke taint indices such as free *m*-cresol, free *o*-cresol, and free phenol in wine with a PCC of about 0.9 (Figure S4A). The correlations are less significant for non-smoke-tainted wine (PCC of 0.6) (Figure S4B).

### Smoke Taint Index Prediction and Smoke-Tainted Wine Classification

**Figure 6:**
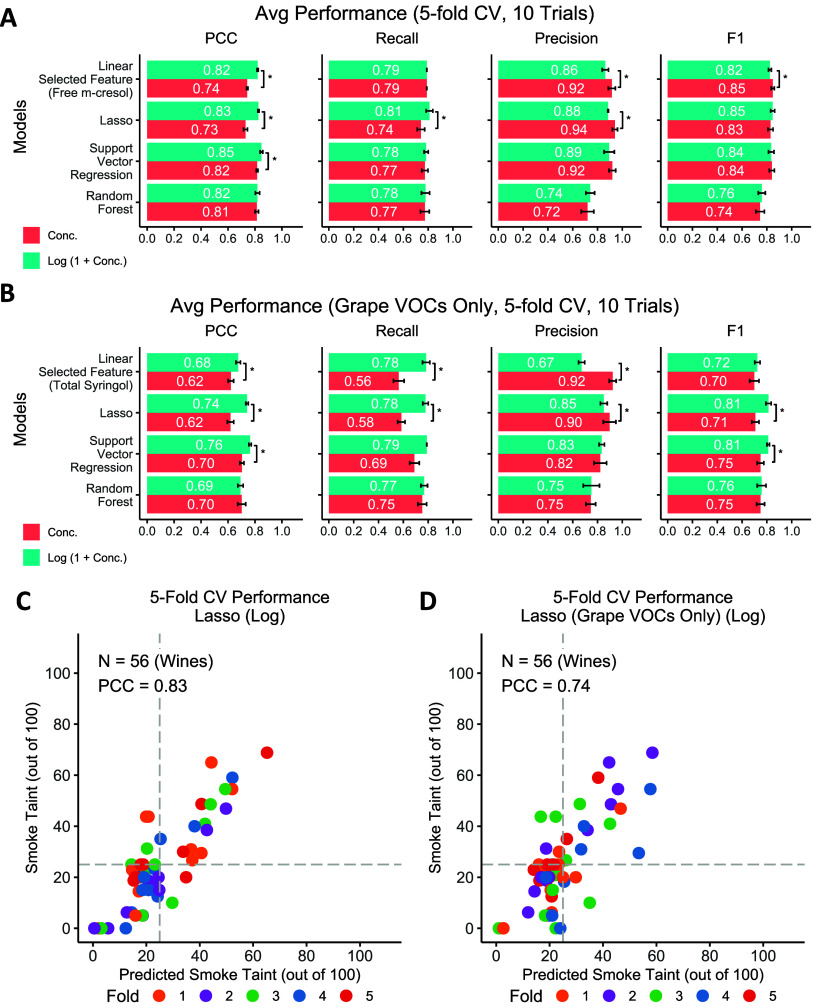
5-fold cross-validation (CV) performance of the models for smoke taint prediction. **A.** Prediction performance of four models using VOCs of wine samples and their origin grape samples as input features. **B.** Prediction performance of four models using VOCs only in the origin grape samples as input features. **C.** Scatter plot of measured smoke taint indices and the indices predicted from the best model (linear model) which used wine VOC concentration (free *m*-cresol concentration) as the input feature. The first trial of the 5-fold CV is shown. **D.** Scatter plot of measured smoke taint indices and the indices predicted from the best model (Support Vector Regression model) which used only VOC concentrations detected in origin grape samples as the input features. The first trial of the 5-fold CV is shown.

**Figure 7:**
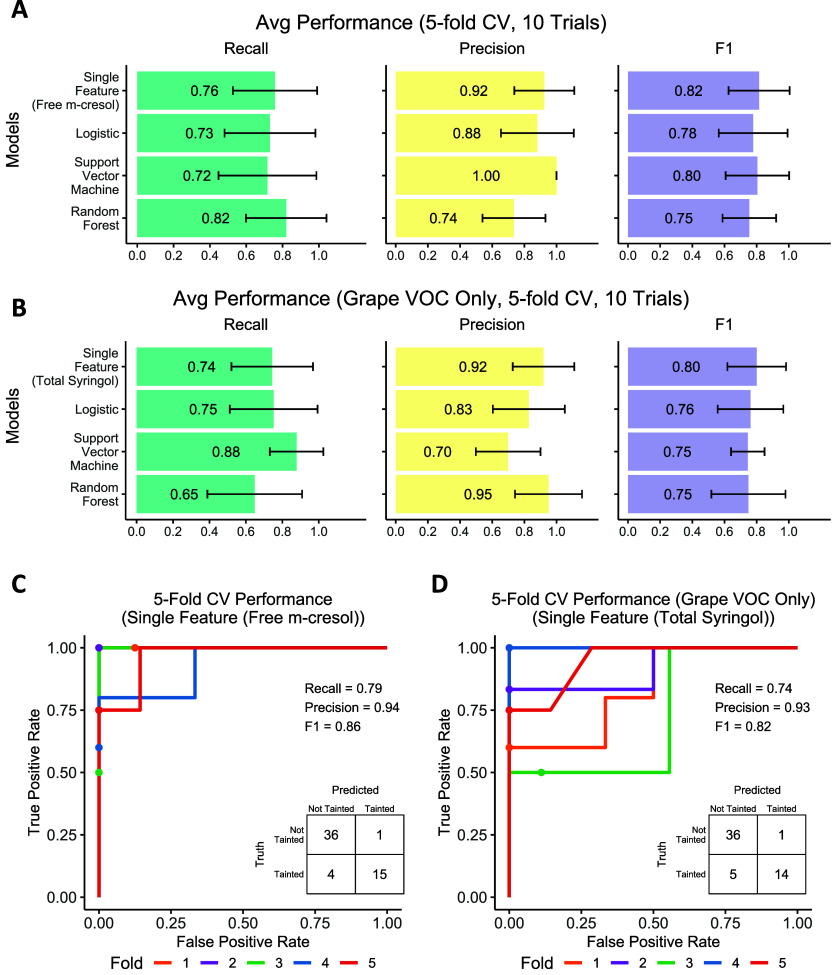
5-fold cross-validation (CV) performance of the models for smoke-tainted wine classification. **A.** Classification performance of four models using VOCs of wine samples and their origin grape samples as input features. **B.** Classification performance of four models using VOCs only in the origin grape samples as input features. **C.** The receiver operating characteristic (ROC) curve of the best model (the single feature model) which used wine VOC concentration (free *m*-cresol concentration) as the input feature. The first trial of the 5-fold CV is shown. **D.** The ROC curve of the best model (the single feature model) which used wine VOC concentration (total syringol concentration in grape) as the input feature. The first trial of the 5-fold CV is shown.

5-fold cross-validation performance of regression models and classification models using selected VOC concentrations (Figure S5) in wine samples and the corresponding origin grape samples as input are evaluated (Figures 6A and 7A). Interestingly, the linear regression model using the most predictive VOCs (free *m*-cresol in wine) as the input feature with log conversion yields the best results with an average PCC of 0.82 in ten 5-fold cross-validation trials, and the single feature classification model yields the best results with an average F1-score of 0.82. In addition, log-converted VOC concentrations yield significantly better performance in three regression models (linear, Lasso, and support vector regression). The performance of models using only VOC concentrations in origin grapes as input features are also evaluated (Figures 6B and 7B): The Support Vector Regression model achieves the best performance with an average PCC of 0.76, and the single feature classification model yields the best results with an average F1-score of 0.80. Log conversion on the VOC concentration also significantly improves the prediction performance in three models.

## Discussion

The data-driven approach discovers more smoke-taint-related attributes of wine such as the origin of smoke-tainted wine, the profiles of VOC concentrations of wine samples and their corresponding origin grape samples, and the correlation between smoke taint index and VOC concentrations, which allow us to find the predictive VOCs of smoke taint index. In addition, the VOC composition ratio changes can be observed by comparing the VOC profiles in grapes and wines. The correlation between VOC concentrations in grapes and wines may help us to discover the difference in metabolism during fermentation in non-smoke-tainted wine and smoke-tainted wine. Although several studies compared the VOC concentrations before and after fermentation,^6,46^ it is difficult to observe the VOC concentration changes for different grape varieties during fermentation due to limited data set size or to correlate the VOC concentrations with smoke sensory attributes without smoke taint index information. In this study, a complete data set that combines VOC concentrations of wine and grape samples from different varieties and smoke taint index is prepared, and it allows us to discover the VOC composition changes and the correlation between VOC concentrations and smoke taint index and to apply predictive modeling of the smoke taint index at the same time. Recently, a study that models the smoke taint flavor based on the VOC concentration in Australian grapes and wine using the Partial Least Squares approach for each variety with listing the VOCs that significantly contribute the smoke flavor is published.^31^ Some VOCs, especially free guaiacol, significantly contribute to or are correlated with the smoke taint flavor in both studies; however, some VOCs are not, such as *m*-cresol. This study which includes the VOC concentration from more varieties of wine and grape samples in California and Oregon may allow us to discover how smoke taint affects the wine quality in different regions and varieties.

The hierarchical clustering results show that smoke-tainted wine samples and non-smoke-tainted wine samples can be separated. The clusters with a significantly higher VOC concentration contain 17 wine samples, and 15 of them are smoke-tainted. In contrast, only 4 smoke-tainted wine samples are clustered into the group with lower VOC concentrations. Although it is reported that several factors such as varieties and maturities of the grapes at smoke exposure can affect the VOC concentrations,^47^ the data set shows that the VOC concentrations in smoke-tainted wine is significantly higher than the non-smoke-tainted wine regardless of other factors.

The correlation analysis results in this study show high correlations between the smoke taint index and the concentrations of specific free VOCs and total VOCs in wine samples, especially free *m*-cresol, *o*-cresol, phenol, guaiacol, *p*-cresol, and 4-methylguaiacol, and total syringol, *o*-cresol, *m*-cresol, 4-methylsyringol, and guaiacol with a PCC greater than 0.65. The results are consistent with the reported results that the ashy sensory attributes are significantly associated with the concentration of free guaiacol, 4-ethylguaiacol, and *m*-cresol^14^ and the observations that glycosidically bound VOCs such as *m*-cresol β-d-glucoside and guaiacol β-d-glucoside significantly contribute the ashy or smoke flavor.^14^ The results also show that free and total *o*-cresol are also highly correlated with the smoke taint index and are also consistent with the low association between the ashy sensory attributes and the concentration of free 4-methylsyringol due to its high detection threshold (10,000 μg/L).^14,48^ However, a high correlation between the smoke taint index and the concentration of total 4-methylsyringol is observed in this study. Further analysis is required to explain the high correlation with the smoke taint index and the concentration of total 4-methylsyringol, but not free 4-methylsyringol. The correlation analysis also shows that the human smoke taint sensor may be saturated as the VOCs concentration increases: The scatter plots of the smoke taint index and VOC concentrations show that the slope is lower when the VOC concentrations increase. Therefore, applying log conversion to the VOC concentration yields a higher linear correlation to the smoke taint index and improves the smoke taint index prediction performance.

The free and total VOCs that highly correlated to the smoke taint index allow us to perform predictive modeling of the smoke taint index. Interestingly, using only log-converted free *m*-cresol concentration as input, the linear regression model achieves the PCC performance of 0.82 and single feature classification achieves the F1-score of 0.82, which argues that even simple models with one or a few markers are sufficient to predict smoke taint. The high PCC between log-converted free *o*-cresol and free phenol may also indicate them as a good predictor as free *m*-cresol. However, they are highly correlated with each other (with pairwise PCC > 0.92), and these VOCs may contribute to the same olfactory sensory receptors related to smoke taint, so combining these features yields no significant improvement in prediction. It is reported that several VOCs including *p*-cresol, *m*-cresol, guaiacol, and 4-methylguaiacol stimulate the similar combination of the olfactory sensory receptors in human or mouse,^49^ and the reported synergistic effect^1,50^ may imply that the different VOCs may trigger the same sensory receptors related to smoke taint and that there is the possibility of changing the smoke flavor intensity irregularly, which increases the difficulties of smoke taint prediction. We expect that as we gather more samples, advanced machine learning models similar to the ones trained here will be able to achieve higher performance for the same features. In addition, the fact that grape-based models achieve a PCC performance of about PCC 0.68 with the use of total syringol as a biomarker demonstrates the capacity for predicting the smoke taint index from grapes before fermentation. It is worth mentioning that free phenol and *p*-cresol concentrations in grapes are negatively correlated with smoke taint but their concentrations in wines are highly correlated with smoke taint, which can be explained by the biodegradation more specifically for phenol and *p*-cresol compared with *m*-cresol and *o-*cresol by *Trichosporon cutaneum*(51) as one of the yeast species involved in wine fermentation.^52^ The VOC composition changes during fermentation may also indicate the degradation of phenol and *p*-cresol in non-smoke-tainted wine: The VOC ratio of free phenol and *p*-cresol is 40% and 20% in grape samples which yields non-smoke-tainted wine, and for these samples, the ratio of phenol and *p*-cresol decreases to less than 20% and 10% after fermentation, respectively. In contrast, a higher composition ratio of free *o*-cresol with a lower degradation rate in yeast is found in grapes which yield smoke-tainted wine. In addition, the increase of the composition ratio of syringol can be explained by the syringol production reported in the previously study^53^ due to bacterial metabolism^54^ (Figure S3). Our results show that producers may accurately use predictive models in either grapes or wine for decision-making when a wildfire event occurs, which in turn can lead to better management and fewer losses.

## Data Availability

The corresponding code of data analysis and predictive modeling of smoke taint in wine is available in the Github repository: https://github.com/IBPA/smoke_taint_prediction.

## Supporting Information Available

Figures including the principal components analysis (PCA) plot of the VOC concentration in the wine samples and the grape samples (Figure S1), the corresponding principal components loadings (Figure S2), the composition ratios of the VOCs in grape and wine samples (Figure S3), the correlation matrices of the VOC concentrations in wine and grape samples (Figure S4), the feature importance represented as the rankings evaluated with different feature selection approaches (Figure S5), and the coefficients of the Lasso models for predicting smoke taint index (Figure S6) (PDF)
