# Supplementary material for: Volatile Organic Compound-Based Predictive Modeling of Smoke Taint in Wine
Source: J Agric Food Chem. 2024 Mar 27;72(14):8060–71. doi: 10.1021/acs.jafc.3c07019 (PMC11010234; doi:10.1021/acs.jafc.3c07019)
Supplement: Supplementary file 1 — jf3c07019_si_001.pdf [file jf3c07019_si_001.pdf]

Volatile Organic Compound-based Predictive Modeling of Smoke Taint in Wine

Cheng-En Tan[1,2,3], Bishnu Prasad Neupane[4], Yan Wen[4], Lik Xian Lim[4], Cristina Medina Plaza[4], Anita Oberholster[4], and Ilias Tagkopoulos[1,2,3*]

[1]Department of Computer Science, University of California, Davis, Davis, California, 95616 United States

[2]Genome Center, University of California, Davis, Davis, California, 95616 United States

[3]USDA/NSF AI Institute for Next Generation Food Systems (AIFS), University of California, Davis, Davis, California, 95616 United States

[4]Department of Viticulture and Enology, University of California, Davis, Davis, California, 95616 United States

[*]Email: itagkopoulos@ucdavis.edu

SUPPLEMENTARY INFORMATION

Supplementary Figures

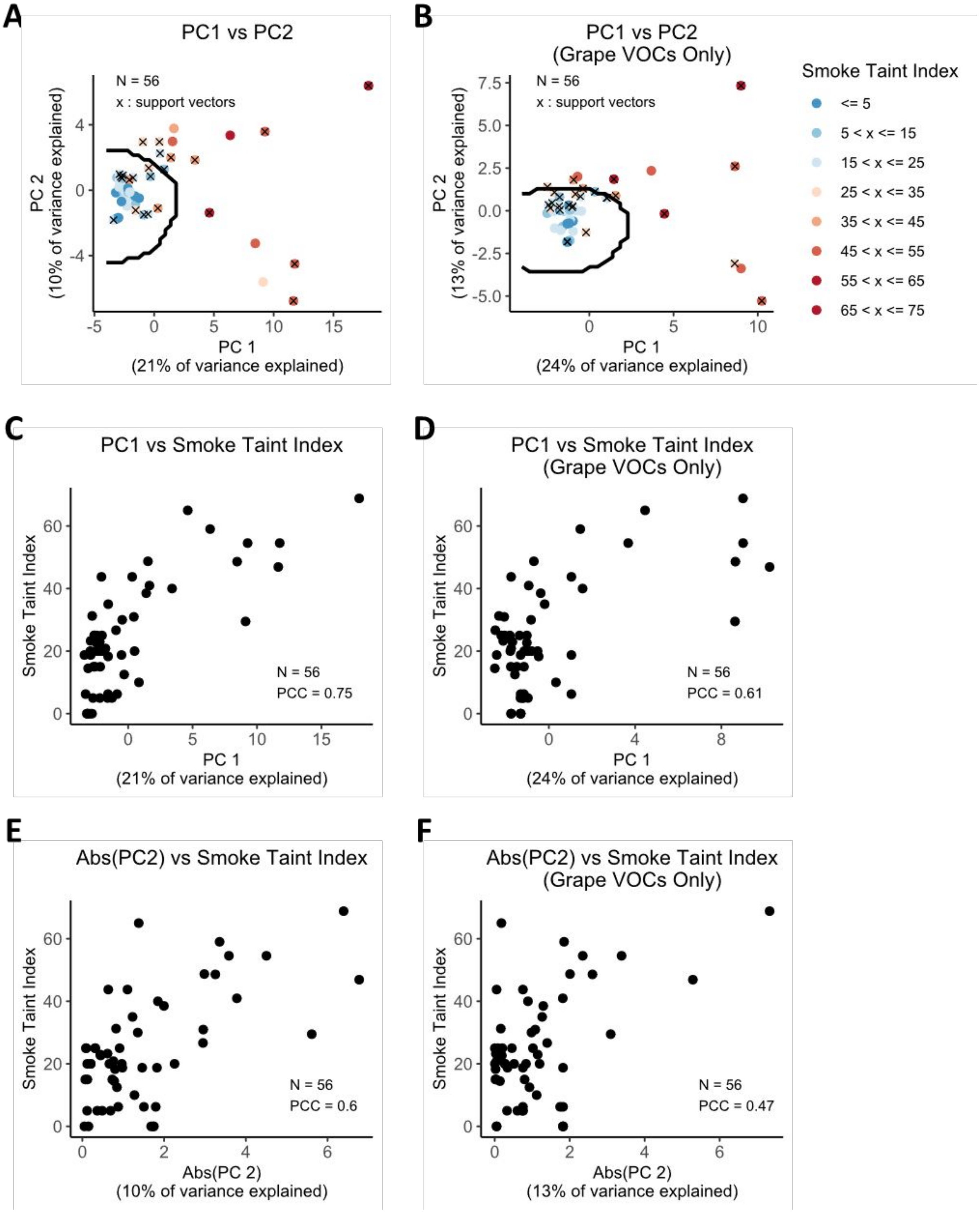

Figure S1. Plots of principal components analysis (PCA). **A.** Scatter plot of principle component (PC) 1 and 2 evaluated from VOC concentrations of wine samples and their origin grape samples. The boundary for classifying smoke-tainted wine (index > 25) is evaluated by the support vector machine model and the support vectors are labeled. **B.** Scatter plots of PC1 and PC2 which are evaluated only from VOC concentrations of the origin grape samples. **C.** Scatter plot of smoke taint index and PC1 (evaluated from both wine and grape VOC concentrations). **D.** Scatter plot of smoke taint index and PC1 (evaluated from grape VOC concentrations only). **E.** Scatter plot of smoke taint index and the absolute value of PC2 (evaluated from both wine and grape VOC concentrations). **F.** Scatter plot of smoke taint index and the absolute value of PC2 (evaluated from grape VOC concentrations only).

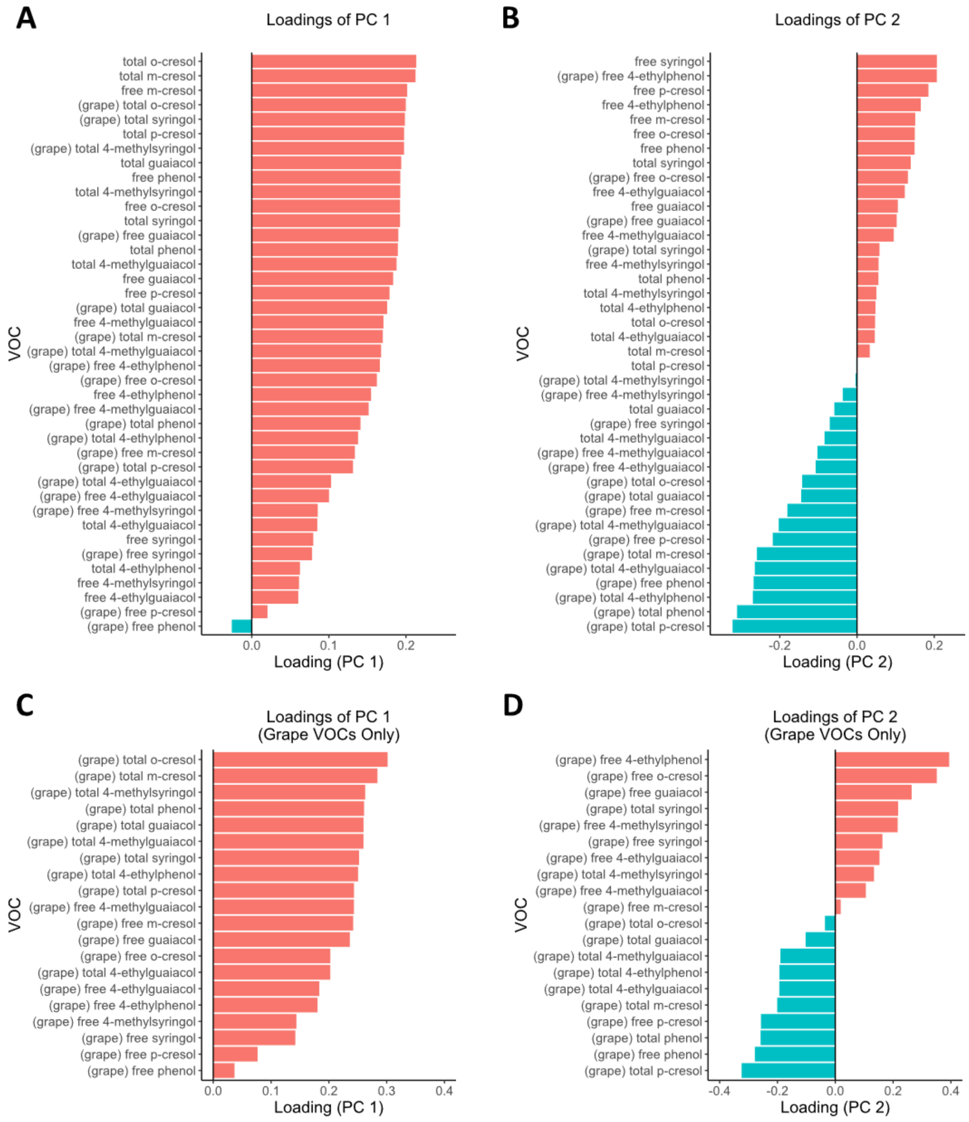

Figure S2. Loading of principle components (PC). **A.** Loadings of PC1 (evaluated from both wine and grape VOC concentrations). **B.** Loadings of PC2 (evaluated from both wine and grape VOC concentrations). **C.** Loadings of PC1 (evaluated from grape VOC concentrations only). **D.** Loadings of PC2 (evaluated from grape VOC concentrations only).

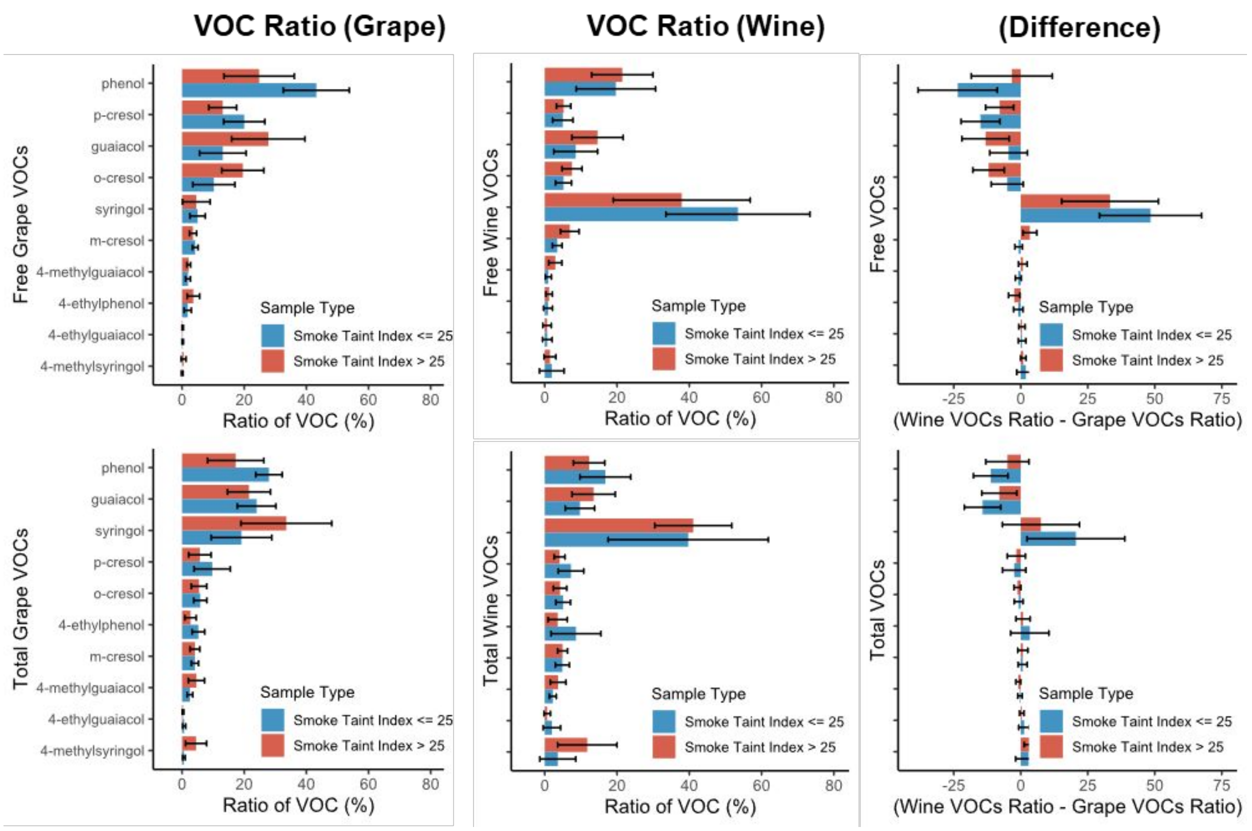

Figure S3. The composition ratios of VOCs in wine samples and their origin grape samples. The difference between the composition ratios of VOCs in wine and grape samples is also shown.

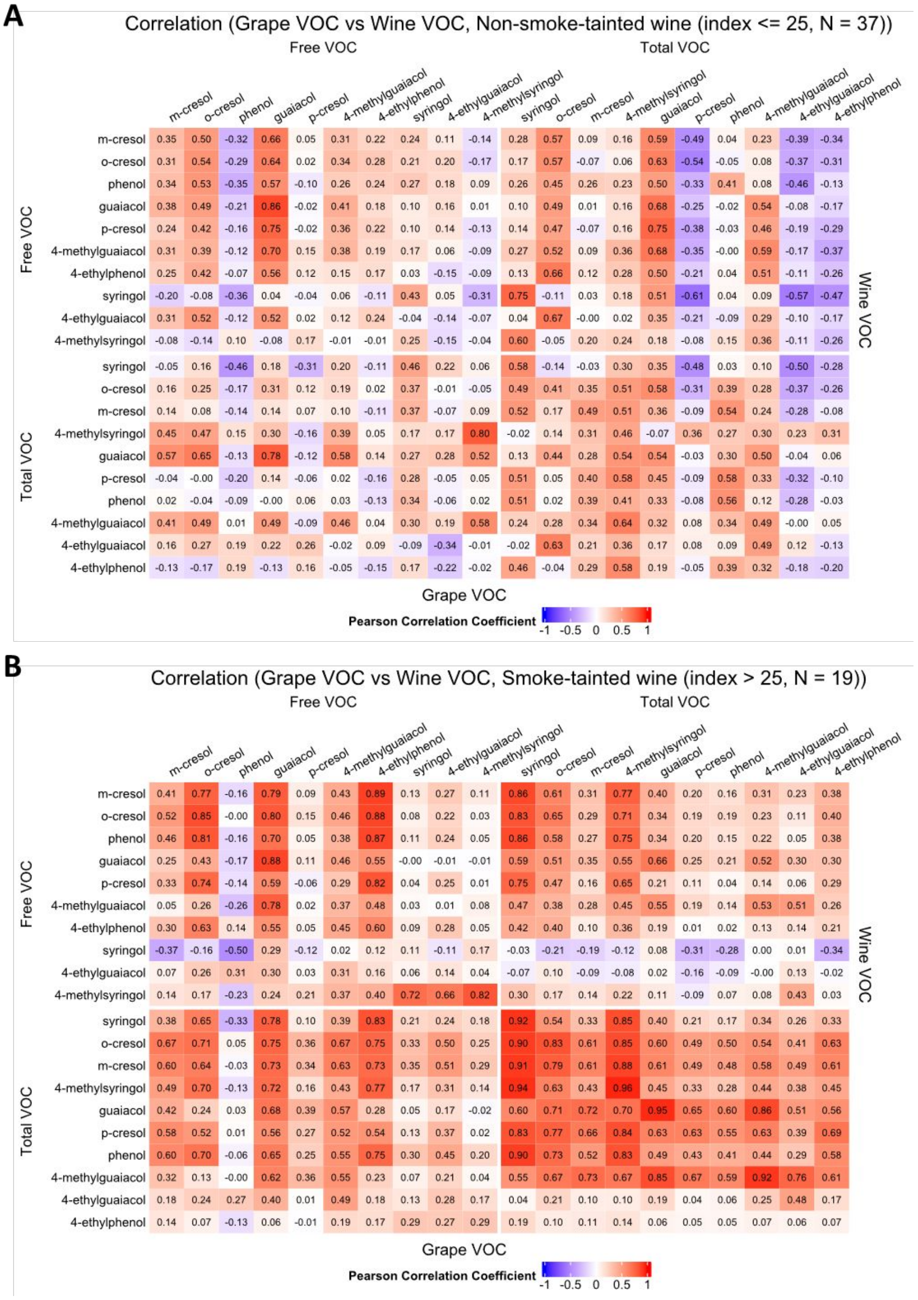

Figure S4. The correlation between the VOC concentrations in wine samples and the VOC concentrations in their origin grape samples. The rows and columns are ordered by the correlation with the smoke taint index. **A.** The correlation matrix for non-smoke-tainted wine (index <= 25). **B.** The correlation matrix for smoke-tainted wine (index > 25).

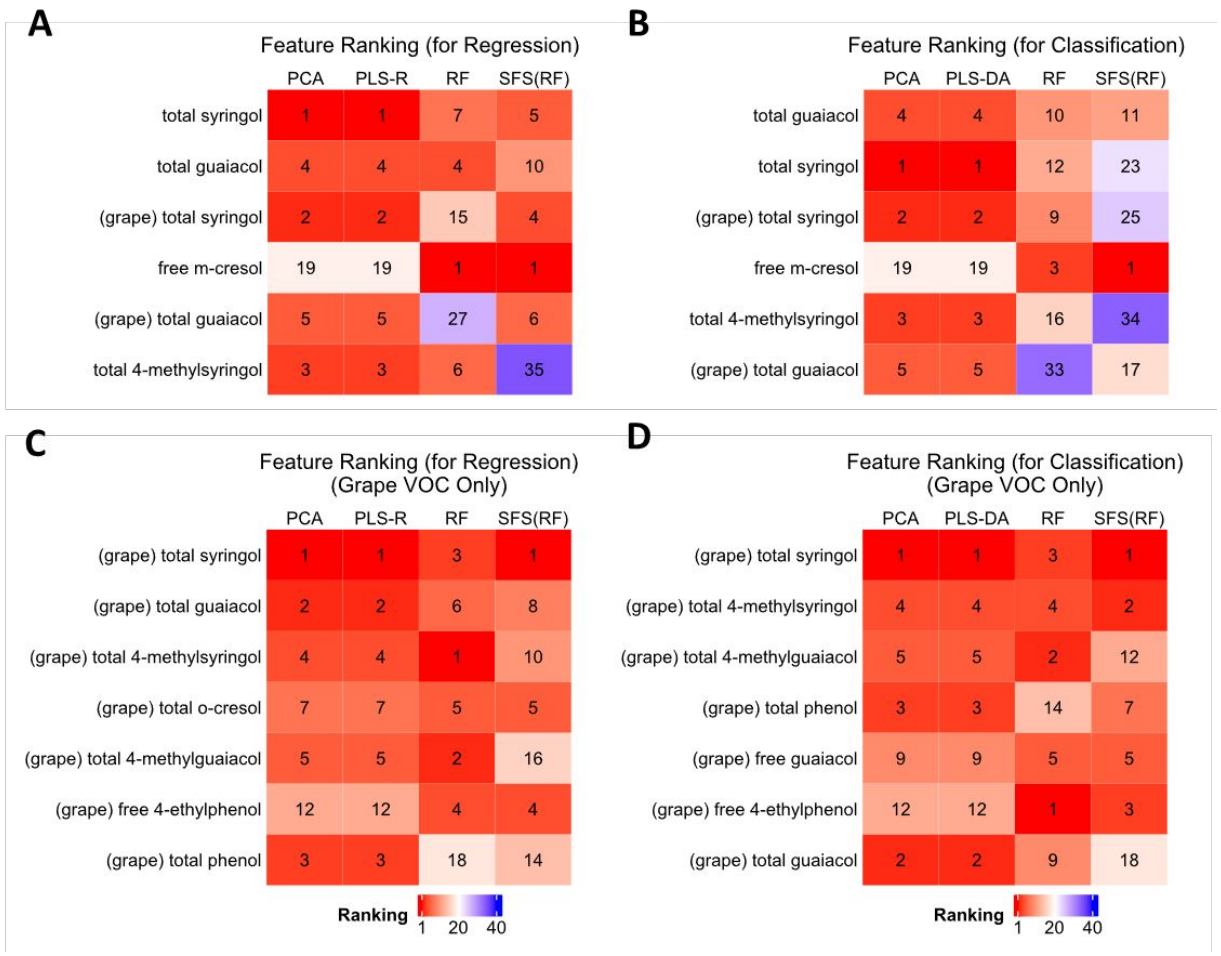

Figure S5. Feature Selection Results. **A.** The selected VOCs for regression which are reported as top five ranking from at least two feature selection approaches. **B.** The selected VOCs for classification which are reported as top five ranking from at least two feature selection approaches. **C.** The selected grape VOCs for regression which are reported as top five ranking from at least two feature selection approaches. **D.** The selected grape VOCs for classification which are reported as top five ranking from at least two feature selection approaches.

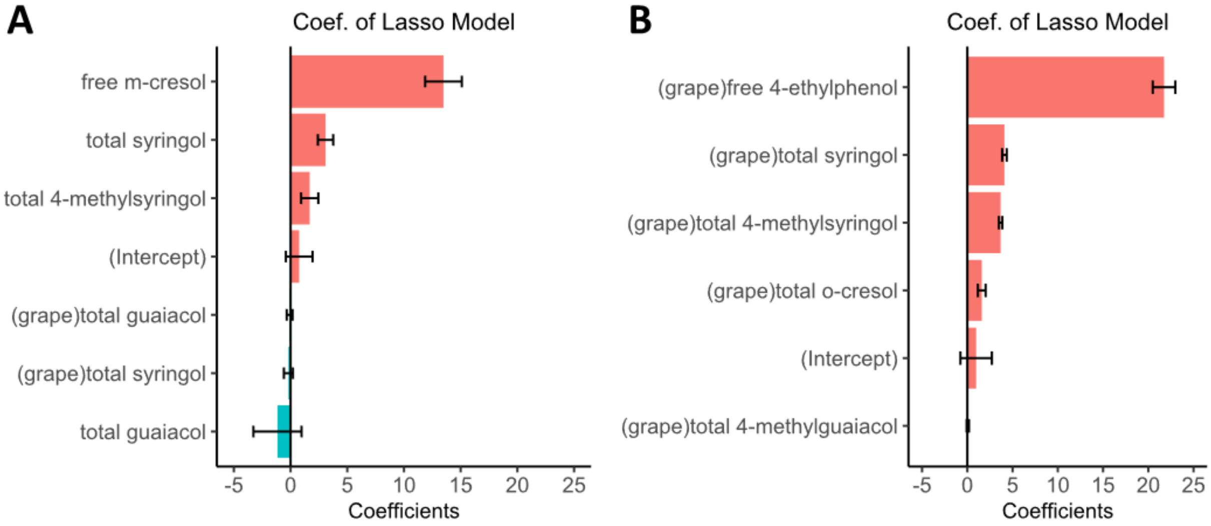

Figure S6. The Coefficient of Lasso models. **A.** The coefficient of the Lasso model using selected wine and grape VOC concentrations as the input features. **B.** The coefficient of the Lasso model using only selected grape VOC concentrations as the input features.
